# Making prisoner deaths visible: Towards a new epistemological approach

**DOI:** 10.1177/26326663231160344

**Published:** 2023-03-06

**Authors:** Philippa Tomczak, Róisín Mulgrew

**Affiliations:** 1 Sociology and Social Policy, 6123 University of Nottingham, Nottingham, UK; 2 School of Law and Irish Centre for Human Rights, 8799 University of Galway, Galway, Ireland

**Keywords:** Detention, suicide, manner of death, cause of death, mortality, homicide, data

## Abstract

In custodial contexts, the duty of states to protect the most fundamental right – to life – is heightened. Nevertheless, prisoner deaths are a universal and frequent concern. The mortality rate among the 11.5 million prisoners globally is up to 50% higher than amongst non-imprisoned persons, forming a human rights and health equity concern. It is therefore peculiar that prisoner deaths have attracted only piecemeal scholarly attention. In this article, we problematize epistemologies of prisoner death, highlighting obfuscations and agglomerations in existing datasets based on poor definitions, reductive statistics and constrained medico-legal categorizations. We provide a springboard towards a new epistemological approach that makes the scale and breadth of prisoner deaths and deceased prisoner characteristics more visible to facilitate prevention. We advance three tenets: *count prisoners who die* rather than deaths in prison, *disaggregate* prisoner death data through rights-informed dimensions and adopt *explicitly defined*, *mutually exclusive categorizations.*

## Introduction

Prisoner deaths are a global and increasing concern (Roulston et al., 2021). Over 11.5 million people are imprisoned globally, of whom 30% have not been convicted (Penal Reform International, 2021: 6). Prisoner mortality rates are up to 50% higher than community rates (UNOHCHR, 2019: 9), forming a global human rights and health equity concern (Winkelman et al., 2022: p.e569). Prisoner deaths are frequently violent, resulting from strangulation, fire, overdose, poisoning, stabbing and homicide (Désesquelles et al., 2018; Ünal et al., 2016). Prisoners remain more likely to die at a younger age than people in the community due to longstanding factors including prison violence, elevated rates of self-inflicted death amongst prisoners relative to the community (Zhong et al., 2021), the prevalence of communicable diseases and inadequate healthcare (Liu et al., 2021). The COVID-19 pandemic also led to elevated prisoner death rates in many countries (Bosworth et al., 2022).

In 2013, the UN Secretary General (paras. 36, 52–56) identified prisoner deaths amongst the biggest challenges related to protecting persons deprived of their liberty. The UN Special Rapporteur on extrajudicial, summary or arbitrary executions recently emphasized that most deaths in custody are preventable (UNOHCHR, 2021). Yet, there is currently no resource that fully illustrates the numbers and causes of prisoner deaths. We simply do not know how many prisoners die each year globally. To reduce prisoner deaths, the UN Human Rights Council recommended in 2019 that states create data management systems to collect, compile and analyse data on prisoner deaths. This article contributes to that valuable and long-overdue project, recommending data tenets to help reduce prisoner deaths.

Collecting, analysing and disseminating accurate death in custody data is ‘critically important for informing public health policies, monitoring […] progress with health development goals’ (Phillips et al., 2014: 1) and underpinning theories about deaths and the effects of intervention (Botchway and Fazel, 2022). Moreover, death data are a prerequisite for monitoring progress towards UN Sustainable Development Goals. Custodial settings are commonly excluded from the service coverage index, masking health inequalities and overestimating progress towards Goal 3.8.1 (Winkelman et al., 2022). Our substantive question is: how can data collection make prisoner deaths more visible and stimulate action?

We problematize epistemologies of prisoner death, highlighting obfuscations and agglomerations in existing datasets based on poor definitions, reductive statistics and constrained medico-legal categorizations. Existing epistemologies underestimate and agglomerate many types of death, of very diverse people, in particular circumstances and locations into just a handful of categories, obfuscating prevention opportunities (Tomczak, 2022). We advocate a new epistemological approach, making the scale and breadth of prisoner deaths and deceased prisoner characteristics visible through more comprehensive and contextualized data.

## Generating a new epistemological approach: methodology and sources

UN calls for action are clear: States should reduce prisoner deaths and create systems that can accurately record how people are dying in prisons. We seek to assist States to achieve these objectives by reconceptualizing data collection to prevent prisoner deaths: requiring exploration and critique of current data systems.

We selected two international datasets on prisoner deaths containing data from multiple countries rather than arbitrarily sampling national statistics. The first dataset is the ‘Mortality in Prison’ section of the UN Crimes Trends Survey (UNCTS) curated by the UN Office of Drugs and Crime (UNODC). The dataUNODC webpage displays available data from 95 countries between 2010 and 2018, highlighting death counts and mortality rates per 100,000 prisoners, alongside counts and rates across manners of death for most listed countries.^1^ There are significant gaps and different countries report in different categories. The second dataset is Table 28 of the SPACE I 2021 statistics: ‘Inmates who died inside penal institutions (during 2020) (numbers, percentages and rates)’ (Council of Europe (COE), 2021: 117–118). These regional statistics are more complete than the UNCTS figures, providing annual numbers and rates of deaths inside penal institutions in COE member States (where available) and demonstrating calculation methods. Data are organized around counts and percentages for homicides, suicides and other deaths, alongside overall mortality and suicide rates per 10,000 inmates per country.

To examine these datasets’ policy-relevance and preventative potential, we analysed multidisciplinary scholarship and prison oversight reports that assess the penological and rights issues relating to prisoner deaths. The ProQuest database was searched to identify all relevant (inter)national scholarships on prisoner deaths. Given our focus on preventable deaths, this search excluded texts on the death penalty, death row, prisoners of war and post-release deaths (see Banwell-Moore, 2022). This scholarship was thematically analysed to identify key issues, themes and knowledge gaps (Petticrew and Roberts, 2006).

We also explored findings relating to prisoner deaths from independent oversight bodies operating in various world regions. A total of 116 reports from three supranational prison monitoring bodies in 58 countries were analysed over the temporal span of their operations: 22 UN Subcommittee for the Prevention of Torture (SPT) country reports (2007–2021),^2^ 80 European Committee for the Prevention of Torture (CPT) country reports discussing 29 countries (1990–2021)^3^ and 14 Inter-American Commission on Human Rights (IACmHR) country reports from 10 countries (1993–2021).^4^ All references to prisoner deaths were analysed, with findings organized thematically around manners/causes of death and controversial aspects. This qualitative, independent dimension formed a vital counterpart to quantitative official statistics, highlighting the often contested nature of prisoner deaths.

Together, our analysis of scholarship and oversight reports represents an extensive (albeit neither exhaustive nor representative) illustration of prisoner death knowledge across different countries and rights systems. Placing data from these diverse sources in dialogue adds rigour, breadth, depth, complexity and richness to our analysis (Denzin and Lincoln, 2017). Insights from these sources produced an evidence synthesis (Popay et al., 2006: 5–6) facilitating informed critiques about the state of prisoner death knowledge and underpinning epistemologies.

Data, categories and concepts are not silver bullets, having complex, often problematic effects*.* We must be careful not to substitute analyses of prisoner deaths for preventative actions. However, a lack of awareness impedes substantive analysis and action*.* First, we explore who and what is invisibilized by contemporary prisoner death data. We then problematize current manners of death in international datasets: natural causes, suicide, homicide and accidental and undetermined/other deaths. The fluid nature and masking effect of these categories are highlighted. We conclude with three new tenets for an epistemological approach that can enhance death prevention.

## What is invisibilized in contemporary data?

Aside from scholarship focused on the death penalty and judicial executions, prisoner deaths have attracted little sustained engagement. Although ‘the ending of life in custody should be controversial’ (Liebling, 2017: 20), prisoner deaths remain ‘sparsely researched’ (Ruiz et al., 2014: 388) and ‘frequent [yet] widely overlooked’ (Gaggioli and Elger, 2017: 35). This is a missed opportunity for safeguarding. Prisoner deaths illustrate the tip of an iceberg of rights and public health issues including ill health, poor quality of prison life and abuse (Tomczak and McAllister, 2021). Wacquant (2010: 199) highlighted how neoliberal prisons ‘invisibilize problem populations … forcing them off the public aid rolls […] and holding them under lock’. However, prisoners are also invisibilized through death, to which sustained and comprehensive attention is long overdue. The relative *invisibility* of prisoner deaths in scholarship, policy and practice may be perpetuating their high and rising numbers. Regarding femicide, Cook et al. (2022: 2) highlight ‘a gap in critical scholarship which interrogates *what* counts as femicide, *who* is invisibilized in official data counts and *how* we should count femicide if we are to […] meaningfully capture the extent of the problem globally’. Prisoner deaths require similar critical examination.

### Prisoner deaths: what is counted?

Because States must rebut the presumption of responsibility for deaths in custody (African Commission on Human and Peoples’ Rights, 2015: para. 37), all prisoner deaths must be investigated (UNOHCHR, 1988: principle 34). Despite the duty to record and investigate prisoner deaths, data illustrating their extent and characteristics are bare. Whilst gross quantifications form only the jumping-off point for understanding this issue, baseline prisoner death datasets are poor globally. Scholars have highlighted absent and unreliable datasets: ‘there are no official data on custodial deaths in China’ (Wu et al., 2020: 1487). Significant undercounting has also been illustrated. In 2015, ‘only 31 of the 97 custodial deaths were reported’ to India's National Human Rights Commission (Ram and Kumar, 2021: 171). In Brazil, Liu et al. (2021) found that the actual number of custody deaths was 2.2 times higher than officially reported. A 2022 US Senate Hearing highlighted failures to count hundreds of deaths in detention annually.^5^ Moreover, the absence of a standard, internationally recognized definition of deaths in custody severely limits capacities to analyse, compare data and act (Ruiz et al., 2014). Addressing this definitional issue is central to making prisoner deaths visible.

We require clarity about which deaths should be counted. The UNCTS counts deaths in all institutions under the prison administration's authority, where persons are deprived of their liberty.^6^ Problematically, this focus on fatalities within prisons excludes prisoners that die following transfer to external medical facilities or those granted temporary leave. SPACE I also focuses on inmates dying inside penal institutions, excluding those dying in public hospitals, during prison leave or whilst absent by permission (COE, 2021: 100). These deaths were excluded from 2018 due to the practical challenges of securing comparable data from all 47 member States (Aebi et al., 2019: 28). This pragmatic epistemological decision to count only deaths occurring within prison facilities diminishes transparency and capacity for evidence-based prevention.

Facility-focused definitions obscure the true scale of prisoner deaths. Daniel (2006: 165) highlighted that US prison suicide victims who die in hospital may be excluded from records. Greek prisoner deaths in 2008 appeared low because the authorities excluded deaths during external hospitalization or temporary release (Cheliotis, 2012). Switzerland's 1984–2000 federal data included deaths outside prison establishments but excluded prisoners who died whilst awaiting trial. This data-gap was ‘unfortunate given the high suicide rates among prisoners awaiting trial’ (Sattar and Killias, 2005: 320).

Such omissions artificially reduce the scale of prisoner deaths reported, diminishing stimulus for reform. The frequency with which prisoners die in hospital, for example, in the end stages of a terminal illness or following resuscitation after a suicide attempt, means this area requires urgent clarification or justification in the UNCTS, SPACE I statistics and national datasets. *Prisoners die outside prisons and these deaths should be counted*. Official data that mask the extent of prisoner deaths do society a disservice. But numbers alone are insufficient: for effective prevention, it is crucial to know who is dying.

### Who is invisibilized in data?

Rights-based data collection approaches require statistics on disadvantaged or marginalized groups at risk of discrimination (UNOHCHR, 2018: 7–8). International datasets are not fulfilling this brief. The UNCTS does not provide *any* disaggregated data. SPACE I is only marginally better, setting out statistics for suicide deaths alone regarding gender (female prisoners) and pre-trial detention (COE, 2021: 112–113). Whilst these datasets are designed to enable comparative analysis from available national statistics, deceased prisoners’ characteristics are being invisibilized.

Although prisoner death is a *gendered phenomenon*, overwhelmingly involving males, this predominance must not obfuscate significant gendered aspects and disproportionalities. Eisler and Smith (2021) highlight the growing number of women dying in USA local jails. Yet gender is entirely absent from the UNCTS data on prisoner deaths and numbers, and in SPACE I, female prisoners are counted only in relation to suicides. There is a dearth of information about transgender prisoner deaths. Whilst transgender prisoners are significantly more likely to experience problems in prison than other populations (Gorden et al., 2017), transgender prisoner deaths are entirely absent from official data. The SPT has highlighted ‘the complete abandonment of transgender women and men in detention’ worldwide, noting ‘deaths of transgender women in custody, including occurrences of death after anal penetration with a bat’ (SPT, 2015: para 68). As such, current datasets provide too little illustration of the gendered nature of prisoner deaths and transgender prisoner deaths are entirely invisible.

Prisons across the world disproportionately hold marginalized groups (Stanley and Mihaere, 2019). Yet dying in prison is stratified by *race and ethnicity*. A total of 75% of prisoner deaths from COVID-19 in New York involved prisoners who identified as Black, Latinx or both (Friedman, 2021). Razack (2015) highlighted how indigenous people disappeared through death in Canadian custodial settings. Cunneen (2006) argued that Aboriginality was frequently a dominant factor leading to incarceration and eventual death in Australia. Race may motivate prisoner–prisoner homicides (Moss, 2006). Prison oversight reports also highlight elevated self-harm and suicide rates in particular categories of prisoners, such as remand (SPT, 2008: para 94) and lesbian (SPT, 2017: para 90) prisoners.

The UN Special Rapporteur on extrajudicial, summary and arbitrary executions highlighted that deaths of imprisoned persons belonging to racial and ethnic minorities ‘reinforce those communities’ experience of systemic and structural racism’, also noting the vulnerability of female and transgender prisoners (UNHRC, 2019: para 40). Although it would be impossible to gather and report data on every attribute of each deceased prisoner in every country, current UNCTS and SPACE I data invisibilize which prisoners are dying. Nuance is required to underpin prevention, particularly for groups already susceptible to discrimination.

### What is visible?

Any ‘death in any type of custody should be regarded as prima facie a summary or arbitrary execution, and there should be a thorough, prompt and impartial investigation to confirm or rebut the presumption’ (*Eshonov v Uzbekistan*, 2010; para. 9.2). Medicolegal death investigations determine one of five *manners of death* (natural, accident, suicide, homicide and undetermined) and the cause of death (a specific injury or disease) (Lunn, 2016). Prisoners die from the same diseases and injuries as the general public. Their deaths are particularly significant, being inextricably entwined with the scale and conditions of imprisonment and ‘potential for death at the hand of another […] or due to neglect’ (Gill and Girela-López, 2015: 402). Yet, ‘it is especially difficult to investigate deaths in prison’ (Smith, 1984: 209). The recording of the manners and causes of prisoner deaths in Greece, for example, has been ‘long beset with vagueness and uncertainty” and “poor recording practices’ (Cheliotis, 2012: 11). State classifications of death may use differing or implicit definitions. Prisoner deaths classified as accidental, suicide, homicide and undetermined are often agglomerated into varying ‘non-natural’, ‘violent’ and ‘external’ classifications.

In categorizing prisoner mortality, the UNCTS adopts a basic dichotomy of ‘natural’ and ‘external’ deaths,^7^ with the external causes category usually further divided into other manners. External deaths are those caused by ‘environmental events or by circumstances suggestive of environmental causes’: including deaths due to intentional injury, accidental/unintentional injury and acute alcohol or drug intoxication but excluding death penalty deaths. UNCTS data are usually presented under four manners/categories: ‘natural causes’, ‘intentional homicide’, ‘suicide’ and ‘accident or other causes’.

COE statistics on inmate deaths in penal institutions include fewer manners of death than the UNCTS. SPACE I refers only to homicide, suicide and ‘other’ deaths. Table 28 of SPACE I provides numbers and percentages for total deaths, homicide, suicide and ‘other’ deaths, alongside mortality and suicide rates per 10,000 prisoners for each country with available data (COE, 2021: 117–119). Data gaps exist due to national inability to disaggregate data across variables: Switzerland noted ‘it is not possible to distinguish death by suicide/homicide/natural causes or by sex’ (COE, 2021: 109). An introductory table ranking COE member States according to core indicators reported only the suicide rate per 10,000 inmates (COE, 2021: 6), obscuring that *the vast majority of prisoner deaths in Europe are categorized as* ‘*other*’, *with no indication of what proportion were potentially preventable*. In 2020, according to median values, 66.6% of deaths in European prisons were classified as ‘other’ (COE, 2021: 118). By providing only total mortality, homicide and suicide counts, these statistics deflect attention from natural, accidental and undetermined prisoner deaths. Whilst these datasets provide valuable information and acknowledge their omissions, the categories used and data collected fail to provide the comprehensive and disaggregated evidence base required for prevention.

## Problematizing prisoner death categorizations

UNCTS data are presented across four manners of death: natural, suicide, intentional homicide and accident and other causes. Insights from scholarship and monitoring bodies highlight their limitations.

### Problematizing ‘natural’ causes

Scholarship and the UNCTS commonly distinguish between ‘natural’ and unnatural/‘external cause’ prisoner deaths. SPACE I subsumes ‘natural’ deaths within ‘other’ causes. Whilst prisoners die from diseases that would have ended their lives irrespective of incarceration (Ruiz et al., 2014), the ‘natural’ prisoner deaths category is deeply problematic, obscuring that many ‘natural’ prisoner deaths are preventable.

Scholarship reveals that a large proportion of prisoner deaths are attributed to natural causes. A total of 88% of deaths in US state prisons were classified as ‘natural’ from 2001 to 2016 (Friedman, 2021). Natural deaths are attributed to ageing, chronic illness (circulatory and respiratory system disease and neoplasms) and infectious and parasitic disease (e.g. Fazel and Benning, 2006). Studies frequently identify cardiovascular disease and cancer as the most common causes of ‘natural’ prisoner deaths (e.g. Jedidi et al., 2018; Ram and Kumar, 2021; Wobeser et al., 2002).

Natural deaths are commonly attributed to ageing prison populations but often ‘reflect serious lapses in healthcare’ (INQUEST, 2020: 5). Razack (2013: 353) highlights how inquests in Canada typically misrepresent the deaths of Aboriginal detainees as *timely*, obscuring violence and representing Aboriginal people ‘as possessing a pathological frailty’ such that ‘each inquest must establish anew how much beyond saving Aboriginal people actually are and how little can be done for them’. Razack's idea of (un)timely deaths has implications for (un)natural prisoner death classifications.

By removing a person's liberty, governments adopt ‘a duty to ensure safe and adequate housing, food, and medical care’ (Gill and Girela-López, 2015: 402). Nevertheless, lack of access to (adequate) medical care too frequently means that prisoners die of existing morbidities at a younger age (Ruiz et al., 2014). Indeed, prisoners are considered ‘older’ at over 50, significantly younger than the community threshold (over 65), due to the ‘accelerated ageing’ experienced in prisons (Greene et al., 2018). Moreover, prisoners are at elevated risk of preventable mortality from communicable or infectious diseases, particularly COVID, HIV/AIDS, tuberculosis and hepatitis C (Bosworth et al., 2022; Lines, 2006), partly due to prison conditions (Rubenstein et al., 2016). COVID-19 produced a large prisoner death toll as prisons form petri dishes for infection due to population density and limited access to protective equipment (Mulgrew, 2022). For all these reasons, it is unhelpful that the ‘natural’ death classification presents deaths as unproblematic, deflecting attention from efforts to identify and address risk factors.

Oversight reports routinely highlight the role of *inadequate prison healthcare in untimely* ‘*natural*’ *prisoner deaths.* In 2005, the CPT noted the deaths of three prisoners, aged 30–43 in Azerbaijan, from non-acute diseases such as pneumonia, raising ‘serious doubts’ regarding ‘the adequacy of the medical care provided’.^8^ In Benin in 2008, the SPT highlighted that most prisoner deaths were from ordinary diseases, including surgical conditions that required hospital treatment.^9^ Lack of prison medical care and lack of access to healthcare staff were also noted.^10^ In Kyrgyzstan in 2012, the SPT noted the high detainee death rate at the country's only penitentiary hospital: catering for 8000 prisoners, this facility had poor sanitation, no central heating, lacked medication and modern equipment, had no psychiatrists and psychologists and was operated by ‘poorly qualified’ and ‘underpaid staff’.^11^

Oversight reports similarly highlight failures to provide *timely hospital treatment*. A female prisoner died in Ukraine in 2005 following a six-day delay in hospital transfer despite suspected and ultimately fatal meningoencephalitis.^12^ A 33-year-old male prisoner died from ‘cardiac insufficiency’ in Azerbaijan in 2006: despite displaying ‘worrying symptoms’ for *months* and presenting as an emergency on the morning of his death, the prison doctor did not call an ambulance.^13^ In 2010, a prisoner in Armenia died requiring medical assistance during the night. It took some time for prison staff to become aware and 45 minutes for an ambulance to arrive.^14^ The CPT reported that there was no regular healthcare staff presence during nights and weekends.

*Security procedures also interrupt timely healthcare*, particularly at night. In Azerbaijan in 2006, two prisoners died in their cells, following a suspected heart attack and an asthma attack, due to cumbersome night-time security arrangements that prevented timely treatment (guards did not have keys and the director's authorization was required to open cells).^15^ A 2010 CPT report highlighted Greek prison authorities’ reluctance to open cell doors at night.^16^ Delays in entering cells in emergencies at night were also recorded in England and Wales in 2020 (Prisons and Probation Ombudsman, 2020: 48).

Misclassification means that non-natural deaths are more frequent than statistics and medical death certification indicate, with a ‘dark figure’ of unrecorded homicides ‘repeatedly confirmed by incidental findings of homicide or even serial murders’ (Madea and Rothschild, 2010: 581). In Romania in 2017, the CPT highlighted a prisoner death that management claimed was from natural causes. The autopsy report concluded the death had been violent, involving ‘a trauma of the skull/brain […] produced most likely by a […] fall […] either self-induced or provoked by another subject/person following an event that has to be the object of an investigation’.^17^ The CPT highlighted their distinct impression that officials at Iaşi Prison did not want to discuss this case. This example also begs the question of who is providing prisoner death figures: prison administrations or independent investigators?

Although ‘natural’ prisoner deaths may appear unconcerning and are absorbed into ‘other deaths’ in SPACE I, this section has highlighted that the ‘natural’ prisoner deaths category is deeply problematic. Simply counting these deaths obscures the unacceptable contexts within which they too frequently occur. Oversight reports show that time and time again, untimely prisoner deaths have resulted from inadequate prison healthcare, failures to provide timely hospital treatment and the risks of locked prison cells, with many prisons rendered structurally unsafe at night.

### Problematizing ‘suicide’

Prisoner suicides are largely avoidable (but see Fenwick et al., 2022), yet account ‘for about half of all prison deaths’ globally (Fazel and Baillargeon, 2011: 959). Preventing self-harm and suicide ‘falls squarely within the mandate of the prison management’ and its duty of care to prisoners.^18^ Yet, national prisoner suicide rates are consistently several times above the general population (Fazel et al., 2017). The USA recorded more than 2000 suicides in local jails alone between 2008 and 2019 (Eisler and Smith, 2021).

Problematically, prisoner suicide is not defined in either the UNCTS 2018 ‘definitions’ or SPACE I, although both sources provide counts and rates. Suicides are typically defined as ‘self-inflicted deaths with the intent to die’ (Stone et al., 2017: 1233). The intention in self-inflicted deaths is often unclear, and confused and mixed intentions are seen in completed and attempted deaths (Walker and Towl, 2016: 31).

Prison suicides are significantly under-reported and may be misclassified. Daniel (2006: 165) highlights underreporting of black inmate suicides in the USA. Regarding Greece, actual suicide rates are artificially reduced through misclassification as accidental or unspecified deaths (Cheliotis, 2012). Moreover, homicides may masquerade or be misrepresented as suicides (Daniel, 2006; Smith, 1984). Hanging is the most common method of prisoner suicide (Gunnell et al., 2005), and deaths by hanging present the greatest risk of insufficient care by the medical examiner (Große Perdekamp et al., 2010). Gill and Girela-López (2015: 404) highlight: ‘Is the hanging death a true suicide or a ligature strangulation homicide made to appear as a suicide? Homicides may be staged to appear as suicides or accidental deaths’.

Suicide methods described in prison oversight reports are diverse and include prisoners dying by banging their heads against walls.^19^ Authorities’ failure to prevent suicide is a recurring theme in oversight reports. In 2015, the CPT found that three prisoners in Ireland died by suicide after the prison authority's failure to (a) effect a referral of a prisoner with a borderline personality disorder and history of self-harm and attempted suicide to a psychiatrist and psychologist, (b) transfer a prisoner with a history of depression and self-harming to a different place of detention to access psychology services following a psychiatrist's request and c) observe a suicidal prisoner in line with medical recommendations following their discharge from hospital after jumping out of a window.^20^

Oversight reports also illustrate the (deliberate) misclassification of homicides as suicides. In Argentina in 2012, the SPT reported that inmates in a facility operating with a ‘palpable climate of brutality and fear at all levels’ alleged that killings had been disguised as suicides.^21^ In 2021 the European Court of Human Rights found that Azerbaijan had failed to protect Alexander Lapshin's right to life: his attempted murder by a group of masked men in September 2017 was recorded by the prison authorities as an attempted suicide (*Lapshin v Azerbaijan*, 2021: paras. 112–113).

Thus, oversight reports demonstrate that simply counting the frequency of ‘suicides’ obscures who is dying, failures to fulfil duties to prevent suicides and failures to keep prisoners safe. The circumstances of these deaths illustrate that many prisoner suicides were likely preventable. Moreover, homicides may be misrepresented and misclassified as suicides, again demonstrating the need for context.

### Problematizing ‘homicide’

Unlike the other categories, the UNCTS defines prisoner deaths by homicide. Intentional homicide is defined in accordance with the UNODC 2016 International Classification of Crime Statistics (ICCS) as an ‘unlawful death inflicted upon a person with the intent to cause death or serious injury’.^22^ This definition has explicit inclusions and exclusions, providing specificity about what should be counted.^23^ In contrast, SPACE I does not define ‘homicide’.^24^

There is remarkably little academic work published in English that engages with prisoner homicide deaths beyond simple counting (e.g. Désesquelles et al., 2018; Jedidi et al., 2018; Wobeser et al., 2002). There are some valuable accounts of individual homicides (e.g. Mashta, 1998) and De Carvalho and Bantim (2019) note 10 homicides during a 2016 riot at Brazil's Agricultural Penitentiary of Monte Cristo. The dearth of prison homicide scholarship is troubling given that: ‘in some countries the rate among prisoners is substantially higher than in the general populations’ (UNODC, 2019: 34). As ‘the State is directly responsible for the well-being of its detainees, high levels of violence and killings within a country's penitentiary system point to a problem that must be addressed at the State level’ (UNODC, 2019: 36). Yet, prisoner deaths have been siloed from broader societal issues: homicide is the most reliable measure of violence, but prisoner violence research seldom considers homicides as an outcome measure (Reisig, 2002: 86).

Oversight reports illustrate various types of prisoner homicides, including prisoner–prisoner violence and lethal/excessive force by prison or state authorities, which are agglomerated in the UNCTS and SPACE I. Although authorities have a responsibility to protect prisoners from others who might cause them harm, prisoners often die in brutal circumstances.^25^ In 2014, the CPT highlighted the death of an infirm elderly man in Ireland following blunt force trauma to his head and trunk, and subsequent cardiac arrest, after his transfer to a shared cell despite a medical recommendation that he be housed alone due to his health and vulnerability.^26^ In 2016 in Brazil, the SPT highlighted (multiple) homicides and a decapitation attributed to severe overcrowding which had exacerbated detainees’ stress levels and competition for resources, resulting in increased prisoner-prisoner violence.^27^

Oversight reports also illustrate staff-prisoner homicides. Inmates died in Cuba following assaults by staff at a maximum-security prison in 2017, where ‘beatings of “handcuffed prisoners” were frequent’. One prisoner died due to a lack of medical care for multiple rib fractures following ‘a major beating inflicted on him for celebrating the death of former President Fidel Castro’.^28^ Prisoners may also die through excessive (lethal) force by police or military authorities. The IACmHR highlighted the killings of 111 prisoners in 1992 during police action following riots in the Carandiru House of Detention in Brazil. Investigations found that military police had riddled naked and defenseless prisoners with bullets in an ‘irrational and indiscriminate use of force’.^29^

The circumstances of prisoner homicides attributable to state authorities may be contested. In Venezuela in 2008, the IACmHR expressed extreme concern after the official version of events stated that a prisoner had died following an escape attempt: the Attorney General concluded that members of the National Guard had simulated punishable action and were responsible for homicide and undue firearm use.^30^ In 2018, the SPT reported a prisoner death in Porto prison, Portugal resulting from a lack of medical assistance following a severe beating from guards during a cell search.^31^ The Portuguese government responded: ‘all deaths of prisoners that occurred in the Porto prison in 2017 and 2018 had natural causes’, arguing that cases referred to the public prosecutor had been closed without criminal proceedings.^32^

While the UNCTS relies on a detailed, precise definition, SPACE I data are compromised by the lack of an explicit definition of what the homicide category includes and excludes. Further, deaths by other prisoners and staff may be misclassified as accidental deaths or suicides. (Intentional) homicide figures agglomerate deaths in different circumstances, obscure significant contributing factors (such as overcrowding) and means of prevention, and conceal responsibility for deaths.

### Problematizing ‘accidental and other’ causes

The UNCTS conflates all ‘accidental and other’ prisoner deaths into one category. The SPACE I statistics are even vaguer: *all* prisoner deaths not classified as homicide or suicide are labelled ‘other’. Much is lost through these agglomerations.

Accidental deaths^33^ happen and the full circumstances of every prisoner death cannot always be known. Yet, remarkably little scholarship published in English engages with the nature and circumstances of accidental and undetermined prisoner deaths, beyond simply describing or quantifying them, for example, Jedidi et al. (2018) found that 7.7% of deaths at Messadine Prison, Tunisia from 2005 to 2016 were categorized as accidental. Accidental and undetermined death classifications are easily overused. The Greek Ministry of Justice systematically fails to provide information about deaths ambiguously classified as ‘found dead’, with *two-thirds of all prisoner deaths* between 1996 and 2000 having *no recorded cause* (Cheliotis, 2012). The accidental and undetermined categories also facilitate misclassification, resulting in underreporting of suicide and homicide (Dooley, 1990).

Oversight reports exemplify problematic and likely preventable deaths. It is frequently hard to establish whether ‘undetermined’ prisoner deaths were deliberate, accidental or self-inflicted. In Bulgaria in 2015, a prisoner died in unclear circumstances. Reports that he died due to accidental major burns from a broken steam heating system were disputed by the Prison Director, who claimed the death resulted from the inmate intentionally damaging pipes.^34^ Thus, oversight reports demonstrate the largely unacknowledged yet problematic nature of agglomerated accidental and other/undetermined death classifications, which obscure details and may facilitate obfuscation of responsibility.

## Death categorizations: fluidity and masking effects

Oversight reports highlight the vast variety of ways in which prisoners can die: a high-profile murder suspect was drowned in a bucket of water,^35^ a prisoner died after being hit in the head with a walking frame,^36^ and another died after a teargas bomb hit his head during the military intervention.^37^ A wide range of (potentially) preventable deaths emerged from oversight report analysis including deaths related to drugs and alcohol, fire, sexual violence, restraints, transmissible disease, prison conditions, inter-prisoner violence, excessive force by prison authorities, and lethal force by state authorities. Yet, datasets conceal these recurring causes of deaths. It is necessary, therefore, to understand how categorizations can obscure common contributors to prisoner deaths and mask responsible and liable actors.

### Fluid deaths, fixed categories

Many prisoner deaths identified in scholarship and oversight reports do not clearly align with the UNCTS categorizations. The frequently disputed nature of deaths and the variety of liable actors means certain deaths can move between manners: deaths related to drugs and alcohol, fire and restraint provide an illustration.

Scholarship highlights the prevalence of *drug and alcohol-related deaths*. Cheliotis (2012) noted that drug overdoses appeared to account for the majority of deaths in Greece, with prescribed and illicit drugs being alarmingly common. In the USA, at least 618 prisoners died in local jails from drug and alcohol poisoning between 2008 and 2019 (Eisler and Smith, 2021). In Irish prisons between 2009 and 2014, positive toxicology for illicit drugs was noted in 26 of 38 unnatural deaths. Illustrating how reliance on manners can conceal important trends, eight of these 26 deaths received a verdict of suicide, 16 were classified as misadventure, and two received an open verdict (Iqtidar et al., 2018). Chronic liver disease caused 16% of prisoner deaths in Texas between 1989 and 2003: these deaths would likely be categorized as ‘natural’ but were underpinned by preventable alcohol abuse and chronic hepatitis B and C infections (Imperial, 2010). Oversight reports similarly document prisoner deaths from drugs,^38^ alcohol,^39^ methadone overdose,^40^ alcohol withdrawal,^41^ and substance withdrawal.^42^ Such deaths may be accidental, result from failure to provide proper care and treatment and/or may be difficult to differentiate from suicide.

The scholarship also highlights the fluid categorization of *prisoner deaths by fire*. In 2011, it was unclear if cell arson deaths in a French prison were due to protest, accident or suicide (Désesquelles et al., 2018). Oversight reports similarly illustrate challenges of categorization. Deaths by fire can be accidental: a prisoner in Azerbaijan died in 2017 from burns after having an epileptic attack while smoking, which led to bedding igniting.^43^ Prison fires can also result from negligence. Following the death of 107 prisoners in a fire in Honduras in 2004, the IACmHR found the authorities had violated the prisoners’ right to life and humane treatment on account of negligence in preventing the fire (*<i>Pacheco Teruel et al. v Honduras*, 2012: para. 60). Deaths by fire may also result from state authorities’ recklessness. In Turkey in 2000, six female prisoners died following a security operation. The autopsy revealed their deaths were fire-related, with the possibility that (the type and number of) devices used by security forces caused the fire or contributed to its spread.^44^ Deaths by fires, resulting from negligence or recklessness, are likely to be categorized as ‘accidental or other’, obscuring both the significance of fire in prisoner deaths and the potential responsibility of authorities.

### Masking effects

The UNCTS categories also conceal contributing factors and knowledge about the victim and perpetrators, masking responsibility and liability and further undermining prevention. Prisoner deaths caused by restraints and sexual violence demonstrate these issues.

In fatal scenarios, the use of *restraints* (in combination with sedation, cuffs and excessive force) can be disproportionate and therefore unlawful.^45^ In 2007, a prisoner died in the Netherlands after being restrained face down in a neck hold, with his ankles and wrists cuffed. The CPT noted that neck holds a risk of positional asphyxia, particularly for prisoners with serious cardiac histories.^46^ In Poland in 2012, a prisoner with alcohol withdrawal died following fixation (due to agitation) for over 35 hours. The CPT highlighted: ‘such a prolonged period of fixation can have no medical justification’.^47^ In Spain in 2013, a 22-year-old prisoner died after being fixated prone (ankles and wrists attached to the bed with straps) for a prolonged period. The CPT noted that this death may have been prevented if restraints had been applied under healthcare supervision and highlighted the risks of using restraints on prisoners who have been given sedatives and neuroleptics (which impair breathing and decrease heart rates).^48^ The CPT called for direct, continuous healthcare staff monitoring of any fixated individual.^49^ Deaths related to restraints and fixation are most likely to be classified as ‘natural’ or ‘accidental and other’, masking the potentially negligent and reckless use of restraints with known fatality risks.

Deaths caused by *sexual violence* are also currently invisiblized. Prisoners may take their own lives following sexual abuse by other prisoners (Pounder, 1986). Oversight reports have highlighted the vulnerability of particular groups of prisoners (transgender, foreign nationals) to forced prostitution and sexual violence that can result in death. In 2013, the SPT reported the death of a 20-year-old in Gabon following ‘a brutal sodomisation’.^50^ Most inter-prisoner sexual violence allegations are unlikely to be reported^51^ and prison staff may be complicit, even paying to watch.^52^ Current categories invisibilize (potential staff involvement in) such deaths, and without provable intent to cause serious injury, such deaths risk being categorized as ‘accidental or other’, limiting awareness and prevention.

Our review of oversight reports demonstrates that current categorizations have significant masking consequences. Prisoner deaths caused (indirectly) by state authorities, including by negligence and recklessness, may be absorbed within the ‘accident and other causes’ category. The ICCS intentional homicide definition includes killings caused by excessive force by law enforcement or state officials but excludes deaths due to legal interventions: those inflicted by law enforcement agencies when suppressing disturbances and maintaining order (UNODC, 2015: 33). Consequently, prisoner deaths caused by legal interventions, often in contested and controversial scenarios, are likely to be counted as accidental or other deaths. Current death data also agglomerate deaths caused by other prisoners, prison authorities or other state agents (police and military), limiting understanding and prevention potential. Further, categorizations do not highlight mass fatalities, implying that each death represents a separate incident and obscuring the potential for mass perpetrators bearing responsibility, whether from gangs, police or military intervention. Accordingly, current categorizations conceal factors (regularly) contributing to death ‘by’ or ‘because’ of prison: the roles played by prison facilities, prison services and penal policies and practices in deaths. Current prisoner death data cannot underpin a comprehensive evidence base and prevention.

## Towards a new epistemological approach

Prisoner death numbers and rates, ideally gathered under explicit definitions, can baldly indicate that certain jurisdictions have higher mortality numbers and rates than others over time, perhaps directing attention and preventative efforts, albeit with significant post-death time lags. International prisoner death data are limited to signalling uncontextualized frequency changes. Even if prisoner deaths were better defined and more consistently reported on, the current epistemological approach under manners of death has limited utility. Fundamentally, the factors that produce prisoner deaths are not illustrated, obscuring points of prevention and impeding policy development.

We have identified limitations of contemporary epistemologies which invisibilize deaths beyond prison walls, characteristics of deceased prisoners, recurring mechanisms of death and liable parties. A more *sophisticated*, *standardized approach* to the collection and reporting of prisoner death data is required nationally, regionally and internationally. Mortality surveillance systems must be capable of underpinning ‘prevention, research, policy, monitoring and evaluation, and allocation of resources’ (Stone et al., 2017: 1233). Answering the 2019 Human Rights Council called for states to collect and analyse up-to-date, comprehensive and disaggregated data about deaths in situations of deprivation of liberty (UNHRC, 2019), we advance three tenets, providing a springboard towards a new epistemological approach that will enable states and inter-governmental bodies to produce more comprehensive, disaggregated and contextualized prisoner death data.

First, we advocate *counting prisoners who die*, rather than deaths occurring within prison facilities. Our broader concept of *prisoner deaths* moves beyond the limitations in international datasets which pragmatically count only deaths *occurring physically within prisons*, obfuscating the true numbers of prisoners who die and preventable deaths. Datasets must recognize that a coercive detention status should supersede the physical location of death. Accordingly, datasets should include *all persons subject to an ongoing detention order* in relation to the criminal justice process (including remand/pre-trial, post-conviction and post-sentence), whether they are physically in a prison facility or outside.^53^ Prisoners that die outside prison facilities, for example, those transferred to hospitals, those die at home following a grant of compassionate leave due to terminal illness and those that die during attempted escapes should be counted.^54^ Whilst including deaths of prisoners on temporary leave is challenging due to variations between national systems, pragmatism should not outweigh transparency.

Our second tenet is to *disaggregate* prisoner death data and examine the *characteristics of prisoners who die*. We have highlighted the obfuscation of prisoner characteristics such as race, ethnicity, sexuality and gender. States should ensure they comply with obligations to collect and publish prisoner death data disaggregated by grounds of discrimination recognized under human rights law. The Office of the High Commissioner for Human Rights highlighted that rights-based approaches to data collection must move away from national averages ‘masking underlying disparities’ to incorporate data on the ‘most disadvantaged or marginalized’ (UNOHCHR, 2018: 7). Agenda 2030 (target 17.18) requires States to produce high-quality, timely and reliable data disaggregated by gender, age, race, ethnicity, migratory status, disability and other relevant characteristics.

Third, it is necessary to *adopt explicitly defined, mutually exclusive categorizations*. The current manners conflate different forms of deaths, with differing causes, levels of responsibility and responsible actors. We demonstrated, for example, how the ‘natural’ causes category masks important distinctions between timely and untimely deaths, whilst the ‘accident and other’ category conflates innocent causes with reckless behaviour. Greater care should be taken to move towards exclusive prisoner death categories with the thorough elucidation of what each includes.

Current data do not provide the detail necessary to develop effective prevention strategies. Additional disaggregation and descriptive tags providing contextual information are required. In relation to intentional homicide, the ICCS provides information about the location of death, the victim, the responsible actor(s) and the means of death, enriching knowledge and helping policymakers identify ‘relevant patterns and trends […] and conduct comprehensive and detailed analyses’ (UNODC, 2015: 15). Adding disaggregating variables on prisoner deaths could enable prison services to implement ‘accurate and responsive evidence-based policies’^55^ and structure data on key analytical grounds rather than medico-legal manners (UNODC, 2015: 7, 12) (Table 1).

**Table 1.** Tenets to guide prisoner death data collection.

Comprehensive	Which deaths?	‘Prisoners who die’ not deaths in prison (persons subject to ongoing detention order), including deaths in external medical facilities; deaths of prisoners on temporary leave; deaths during escape; deaths of sentenced prisoners; deaths of pre-trial detainees; deaths of prisoners awaiting sentence
	How did the prisoner(s) die?	Manners of reported deaths must be: - Detailed (reduce reliance on ‘other’; ensure all major forms of death represented) - Distinct (reduce overlap between manners) - Specific (clear definitions and boundaries) - Verified (submitted by an independent body and the source made clear)
Disaggregated	Which prisoners died?	Extend data to include a broader range of rights-required identifiers: - gender (identity) - race, ethnicity - age - sexuality - disability - legal status etc.
Contextualized	Who or what was involved in the death?	Available information that would enhance death prevention potential should be added, including - Tags to indicate if the death was related to drug and/or alcohol misuse, fire, sexual violence, restraint etc. - Where did the death occur? Who was responsible for the death? - In what way were they responsible (negligence, recklessness, intentional)? Was the death part of an incident resulting in mass fatalities? Were weapons used?

In addition to these data collection adjustments, it is important to recognize that cumbersome, manual and decentralized information recording procedures also impact data quality, transparency and accessibility (PRI, 2021: 40). Our suggestions require the *digitalization of data collection*. Digitizing data collection through automated electronic data capture, storage, transfer and compilation significantly improves (inter)national abilities to capture and retrieve disaggregating variables and higher detail (UNODC, 2015: 15). Digitalization is occurring in other mortality statistics: the latest WHO *International Classification of Diseases* for systematic recording, reporting, analysis, interpretation and comparison of mortality data is fully electronic.^56^ While digitalization requires investment, access to sophisticated, standardized systems that collect comprehensive, disaggregated prisoner death data would enable the monitoring of progress towards the Sustainable Development Goals.^57^

To achieve these objectives and the reforms advocated by various UN bodies, the UNCTS approach to collecting and disseminating mortality in prisons data should be modified. The UNODC should be tasked with conducting this revision and facilitating the necessary consultations with relevant, interested and expert stakeholders. The UNODC should also identify, provide and coordinate any support or technical assistance required by States to operationalize this revised approach to prisoner death data collection, particularly in light of the obstacles posed by the digital divide.

## Conclusion

Prisoner deaths clearly require more academic and policy attention to uphold the rights of individual prisoners and facilitate broader reform. Illustrating the ‘tip of the iceberg’, prisoner fatalities indicate the state of rights, health and safety within prison systems. Existing epistemologies disguise the scale of the issue, invisibilize prisoner characteristics and obscure the circumstances of deaths. We problematized the agglomeration of many deaths into a handful of categories that obfuscate prevention and forms of responsibility. We highlighted the risks of miscategorization across the manners of death, which conceal the avoidable nature of many prisoner deaths, points of prevention (such as ensuring timely hospital treatment for unwell prisoners), contextual contributors (such as difficulty entering cells at night, overcrowding) and prison systems’ failures to fulfil duties such as preventing suicide, potentially obfuscating state responsibility.

The current UN spotlight on prisoner death prevention provides an important opportunity for scholars and NGOs to remove the policy ‘blind spots’ created by current epistemologies. Moreover, political willingness to engage with scientific calls for prison policy reform and greater data transparency following COVID-19 should be harnessed. The now ‘medicalized landscape of prison reform’ makes requests for increased data sharing and transparency more palatable (Jain, 2020: 680). This alignment of international prioritization and increased political willingness nationally creates a unique moment for reimagining prisoner death data and revising the UN approach to collecting prisoner death data.

We make recommendations for substantive reconsideration of prisoner death epistemologies to produce an enhanced evidence base facilitating prevention. Our tenets will help to overcome the masking effects of current categorizations and help unlock the protective value of data, generating means of disrupting constrained epistemologies by facilitating accurate, comprehensive and disaggregated prisoner death data. Introducing more sophisticated, standardized and digitalized prisoner death data collection systems would help authorities to harmonize data collection, identify patterns and trends, improve prison monitoring, implement international standards and monitor progress towards the Sustainable Development Goals.^58^ Our recommendations will empower authorities to produce a rights-informed evidence base for policies and practices that reduce prisoner deaths and improve the quality of life in prisons.

## Notes

1. See https://dataunodc.un.org/data/Prison/Mortality%20in%20prison.

2. Argentina, Benin, Bolivia, Brazil, Chile, Honduras, Kyrgyzstan, Mexico, North Macedonia, Mali, Niger, Peru, Portugal, Paraguay, Sweden, Togo, Ukraine, the United Kingdom and Uruguay.

3. Armenia, Austria, Azerbaijan, Bosnia and Herzegovina, Bulgaria, Cyprus, Estonia, Georgia, Greece, Hungary, Iceland, Ireland, Italy, Kosovo, Latvia, Lithuania, Malta, North Macedonia / Former Yugoslav Republic of Macedonia, Norway, Poland, Portugal, Romania, Serbia, Slovenia, Spain, The Netherlands, Turkey, Ukraine and the United Kingdom.

4. Brazil, Colombia, Cuba, Dominican Republic, Ecuador, Guatemala, Haiti, Honduras, Peru and Venezuela.

5.
https://www.hsgac.senate.gov/imo/media/doc/2022-09-20%20PSI%20Staff%20Report%20-%20Uncounted%20Deaths%20in%20America's%20Prisons%20and%20Jails.pdf

6. See the Data questionnaire ‘definitions’ section at https://www.unodc.org/unodc/en/data-and-analysis/statistics/crime/cts-data-collection.html.

7. See the data questionnaire ‘definition index and counting units’ section at https://www.unodc.org/unodc/en/data-and-analysis/statistics/crime/cts-data-collection.html

8. CPT/Inf (2017) 11, para. 46.

9. CAT/OP/BEN/1, para. 222.

10. Ibid, para. 215.

11. CAT/OP/KGZ/1, para. 91.

12. CPT/Inf (2007) 22, para. 125.

13. CPT/Inf(2017) 12, para. 93.

14. CPT/Inf (2011) 24, para. 100.

15. CPT/Inf (2017) 11, para. 46; CPT/Inf(2017) 12, paras. 64, 93.

16. CPT/Inf (2010) 33, para. 129.

17. CPT/Inf (2019) 7, para. 76(iii).

18. CPT/Inf (2020) 18, para. 107.

19. OEA/Ser.L/V/II.1111 Doc.21 Rev 6, para. VIII(30).

20. CPT/Inf (2015) 38, para. 29.

21. CAT/OP/ARG/1, para. 77.

22. See Offence 0101 Intentional Homicide, Definitions section, UNCTS Data Questionnaire File at https://www.unodc.org/unodc/en/data-and-analysis/statistics/crime/cts-data-collection.html.

23. For example, murder, serious assaults leading to death and deaths caused by extrajudicial killings are included, whereas deaths caused by self-defence, involuntary or non-negligent manslaughter are not. See UNODC, 2015: Table 3 at 33.

24. States rely on definitions in their national penal codes.

25. CAT/OP/GAB/1, para. 58; CPT/Inf (2002) 26, para. 72.

26. CPT/Inf (2015) 38, para. 29 (ii).

27. CAT/OP/BRA/3, para. 50.

28. OEA/Ser.L/V/II. Doc.2/20, para. 375.

29. OEA/Ser.L/V/II.97 Doc.29 Rev.1, para. IV (35).

30. OEA/Ser.L/V/II. Doc.54, paras. 883.

31. CAT/OP/PRT/1, para. 57.

32. CAT/OP/PRT/1/add.1, para. 31.

33. Death within 30 days as a result of an injury accident” (Crump et al, 2013: 297). Accidents can include falls, accidental poisoning (alcohol, narcotics, drugs and chemicals) or transport incidents.

34. CPT/Inf (2018) 15, para. 69.

35. CPT/Inf (2008) 31, para. 28.

36. CPT/Inf (2019) 7, para. 76.

37. CPT/Inf (2001) 31, para. 16.

38. CPT/Inf (2009) 25, para. 71.

39. OEA/Ser.L/V/II.1111 Doc.21 Rev 6, para. VIII(32).

40. CAT/OP/MKD/1, para. 28; CPT/Inf (2009) 13, para. 48

41. CPT/Inf (2015) 40, para. 45.

42. CPT/Inf (2008) 7, para. 79.

43. CPT/Inf(2017) 12, para. 65.

44. CPT/Inf (2001) 31, para. 15.

45. CPT/Inf (2005) 13, para. 17.

46. CPT/Inf (2012) 21, para. 106.

47. CPT/Inf (2014) 21, para. 90.

48. CPT/Inf (2013) 8, para. 16.

49. CPT/Inf (2013) 8, para. 17.

50. CAT/OP/GAB/1, para. 57.

51. CPT/Inf (2008) 2, para. 45.

52. See CAT/OP/PRY/1, 07.06.10, para. 214.

53. For SPACE I, Romania counts all persons that died during a prison sentence, including those that died outside prisons (COE, 2021: 108-109).

54. Deaths after permanent sentence suspension under compassionate release policies fall outside our definition but create a closely associated (large) group of deaths in some jurisdictions. For example, relatively low recorded prisoner death numbers in Tunisia are likely due to common sentence suspension for terminally ill prisoners (Jedidi et al, 2018).

55. UNODC, The UN Survey on Crime Trends and the Operations of Criminal Justice Systems, 2.

56. WHO, ICD-11 Fact Sheet, 1.

57. UNODC, The UN Survey on Crime Trends and the Operations of Criminal Justice Systems, 1-2.

58. A/76/264, para. 65.
